# Screening and Psycho-Oncological Support for Patients With Head and Neck Cancer and Brain Malignancies Before Radiotherapy With Mask Fixation: Results of a Feasibility Study

**DOI:** 10.3389/fpsyg.2021.760024

**Published:** 2021-12-16

**Authors:** Sebastian Adeberg, Christina Sauer, Lena Lambert, Sebastian Regnery, Paul Windisch, Karim Zaoui, Christian Freudlsperger, Julius Moratin, Benjamin Farnia, Christoph Nikendei, Juergen Krauss, Johannes C. Ehrenthal, Rami El Shafie, Juliane Hörner-Rieber, Laila König, Sati Akbaba, Kristin Lang, Thomas Held, Stefan Rieken, Juergen Debus, Hans-Christoph Friederich, Imad Maatouk

**Affiliations:** ^1^National Center for Tumor Diseases (NCT), University Hospital Heidelberg (UKHD) and German Cancer Research Center (DKFZ), Heidelberg, Germany; ^2^Department of Radiation Oncology, University Hospital Heidelberg (UKHD), Heidelberg, Germany; ^3^Heidelberg Institute for Radiation Oncology (HIRO), National Center for Radiation Research in Oncology (NCRO), UKHD and DKFZ, Heidelberg, Germany; ^4^Clinical Cooperation Unit Radiation Oncology, German Cancer Research Center (DKFZ), Heidelberg, Germany; ^5^Department of General Internal Medicine and Psychosomatics, University Hospital Heidelberg (UKHD), Heidelberg University, Heidelberg, Germany; ^6^Department of Radiation Oncology, Kantonsspital Winterthur, Winterthur, Switzerland; ^7^Department of Otorhinolaryngology, Head and Neck Surgery, University Hospital Heidelberg, Heidelberg, Germany; ^8^Department of Oral and Maxillofacial Surgery, University Hospital Heidelberg, Heidelberg, Germany; ^9^Department of Radiation Oncology, Sylvester Comprehensive Cancer Center, University of Miami, Miami, FL, United States; ^10^Department of Psychology, University of Cologne, Cologne, Germany; ^11^Department of Radiation Oncology, Goettingen University Hospital, Goettingen, Germany; ^12^Section of Psychosomatic Medicine, Psychotherapy and Psychooncology, Department of Internal Medicine II, Julius-Maximilian University Würzburg, Würzburg, Germany

**Keywords:** cancer, radiation, mask fixation, distress, head and neck

## Abstract

This single-center, single-arm trial investigates the feasibility of a psycho-oncological care program, which aims to reduce psychological distress and improve compliance with radiotherapy with mask fixation in patients with head and neck cancer or brain malignancies. The care program comprised (1) a screening/needs assessment and (2) the provision of a psycho-oncological intervention using imaginative stabilization techniques for distressed patients (distress due to anxiety ≥5) or in a case of subjective interest in the psycho-oncological intervention. Another allocation path to the intervention was directly through the radiation oncologist in charge who classified the patient as: in need of support to tolerate the immobilization device. Of a total of 1,020 screened patients, 257 (25.2%) patients indicated a distress ≥5 and 141 (13.8%) patients reported panic attacks. 25% of the patients reported a subjective interest in psycho-oncological support. A total of 35 patients received the psycho-oncological intervention, of which 74% were assigned by radiation oncologists. In this small patient cohort, no significant pre-post effects in terms of depression, anxiety, distress, and quality of life (mental and physical component scores) could be detected. Our results indicate a good feasibility (interdisciplinary workflow and cooperation, allocation by physicians in charge) of the psycho-oncological care program for this cohort of patients before radiotherapy with mask fixation. The screening results underline the high psychological distress and demand for psycho-oncological support. However, since the utilization of our intervention was low, future studies should reduce the barriers and improve compliance to psycho-oncological services by these patients.Clinical Trial Registration: https://www.drks.de/drks_web/setLocale_EN.do #DRKS00013493

## Introduction

Up to 60% of the patients with head and neck cancer (HNC) or brain cancer (BC)/secondary brain malignancies experience increased levels of distress, anxiety, and depression (Kilbride et al., 2007; Penner, 2009; Cordes et al., 2014; Mackenzie et al., 2014; Reich et al., 2014). This prevalence is higher when compared to that in other entities (Pascoe et al., 2004; Epstein et al., 2005; Dilworth et al., 2014). It has also been reported that 41% of HNC patients are diagnosed with a psychological disorder (Mehnert et al., 2014), which is the second highest prevalence among cancer entities after patients with breast cancer (42%). Majority of the HNC patients who receive treatment with radiotherapy are secured with a fixation mask during radiotherapy. Patients experience wearing the mask as distressing (Oultram et al., 2012) and as the worst part of the treatment (Rose and Yates, 2001). It is also documented that 26% of HNC patients are impacted by “mask anxiety” (Moschopoulou et al., 2018), which encompasses feelings of distress, anxiety, and claustrophobia (Nixon et al., 2019). The fitting and first treatments elicit elevated levels of psychological distress (Nixon et al., 2019; Keast et al., 2020). The impact on the patients’ distress level has a high chance of increasing treatment interruptions (Clover et al., 2011; Oultram et al., 2012). Among HNC survivors, one-third shows subclinical cancer-related post-traumatic stress symptoms 2 years since cancer treatment (Moschopoulou et al., 2018). Against the background of the increasing number of HNC survivors (Pulte and Brenner, 2010; Schmidt Jensen et al., 2018) and increasing survival rates of lung cancer patients (Antonia et al., 2017) with a high probability of developing brain metastases, consideration of psychological distress is not only important during cancer treatment, but also in long term. The implementation of distress screening is an economic way of detecting emotional burden and needs and offering psycho-oncological support; however, it is not routinely performed so far.

Recommendations for physicians managing HNC patients for different treatment phases share the goal of maintaining compliance to radiotherapy (Reich et al., 2014). In analogy to radiotherapy, data for radiologic diagnostics are available. Here, claustrophobic fears and anxiety led to more frequent disruptions of procedures (Harris et al., 1999; Hollenhorst et al., 2001). High-quality images with 1mm slice thickness for stereotactic radiotherapy planning can lead to examination times of over 30min and the necessity for repetitive examinations (25). However, there is a robust evidence that the mask fixation and mask anxiety are one of the major constraints, as well as a compliance-restricting factor during radiotherapy (Rose and Yates, 2001; Clover et al., 2011).

In clinical practice, the assessment and consideration of the patients’ needs and psychological burden before mask fixation are worthy of improvement (Halkett et al., 2010). Only a limited number of studies have addressed specific patient-related psychosocial variables and needs of individuals undergoing radiotherapy of the brain or head and neck region (Clover et al., 2011; Nixon et al., 2018). They show an increase in depression but mixed evidence regarding general anxiety (Rose and Yates, 2001; Kohda et al., 2005; Chen et al., 2009). Addressing psychosocial demands and assessing psychological symptoms early in the course of a disease and pretreatment can positively influence multiple patient-related factors, including compliance to radiotherapy (Gold, 2012). Multidisciplinary team care, including a psychosocial screening and integrated psycho-oncological support, may help to decrease psychological distress and increase the compliance of patients undergoing radiotherapy with mask fixation (Williams, 2017).

Psycho-oncological interventions show positive effects on emotional distress and quality of life (QoL) in cancer patients (Faller et al., 2013). In patients with HNC, cognitive behavioral therapy (CBT) and behavioral medicine (e.g., relaxation training and hypnosis) are promising; however, the literature is scarce (Williams, 2017). Recently, two separate case studies described the efficacy of CBT intervention (Dabrowski and Grayer, 2018) and eye movement desensitization and reprocessing (EMDR; Dinapoli et al., 2019) for HNC patients to reduce anxiety and increase compliance to their potentially life-saving treatment. However, studies with larger samples are needed to investigate the feasibility and efficacy of those treatments.

Imaginative stabilization techniques (IST) represent another promising approach for increasing relaxation and emotion regulation for patients undergoing mask fixation and radiotherapy. The practice of IST offers a low-threshold time-effective psycho-oncological support for patients with increased anxiety and distress (Luebbert et al., 2001; Roffe et al., 2005). Patients can use IST as a skill to gain control over overwhelming feelings in threatening situations [e.g., mask fixation, magnetic resonance imaging (MRI), and to detach from the situation (Rosenberger, 2016)]. Recent studies show that patients intuitively use visualization/IST as a skill during mask fixation and radiotherapy (Nixon et al., 2019; Keast et al., 2020). However, so far, no study has investigated the implementation of IST in patients before radiotherapy.

Against this background, we developed a psycho-oncological care program for patients with HNC or brain malignancies before radiotherapy with mask fixation. The program comprised (1) a screening for all patients with HNC or brain malignancies before radiotherapy with mask fixation and (2) provision of a psycho-oncological intervention using IST for distressed patients (indicated by screening or radiation oncologists).

This trial aimed to examine the feasibility of the developed care model, that is, (1) feasibility of the screening, which entails the following: integration in the clinical routine (feasibility of the screening as part of the registration); interdisciplinary workflow and cooperation (data transfer from the Department of Radiotherapy Oncology to the psycho-oncological service); number of completed screenings; extent of psychological distress and demand for psycho-oncological support indicated by the screening (in the sense of a needs assessment); and reasons for non-participation in the intervention.

It also examined (2) the feasibility (utilization, acceptance, and retention of radiotherapy) of a psycho-oncological intervention using IST to reduce anxiety and improve compliance to radiotherapy with mask fixation. Our clinical outcomes (secondary outcomes) are psychological distress, depression, anxiety, and QoL.

## Materials and Methods

We performed a feasibility study to examine a psycho-oncological care program for patients with HNC or brain malignancies who are starting treatment with radiotherapy and mask fixation at the Department of Radiation Oncology, University Hospital Heidelberg, Germany. The care program comprised (1) screening and (2) provision of psycho-oncological intervention. From November 2017 till June 2019, all patients aged >18years were screened with a 3-item self-developed measurement tool. The screening tool contained items that ask question about the distress that arises due to anxiety caused by the medical examinations, e.g., MRI (with a Likert scale of 1 to 10; 1=not distressed; and 10=extremely distressed), panic attacks (yes/no) during the last 4weeks (to evaluate the intensity of anxiety in distressed patients), and (in 50% of patients) subjective interest in a supportive psycho-oncological intervention. The last item was added to also reach out to patients with interest in the intervention without being distressed. All patients at all stages in the course of the disease received the screening during registration as part of the clinical routine at the outpatient clinic of the Department of Radiation Oncology and completed the screening subsequently after their first visit. Once a week, a study team member collected and evaluated the screening forms. All patients with a distress score≥5^1^ or subjective interest were contacted by a psycho-oncologist through a phone and offered participation in the psycho-oncological intervention. Another allocation path was by the radiation oncologist in charge who identified the patient as “in need of support” to carry out radiotherapy with a mask-fixation independent of the screening score. The inclusion criteria for the intervention study were as: (1) the presence of HNC, BC, or brain malignancies; (2) planned radiotherapy with mask fixation; (3) high psychological distress indicated by screening or the physician in charge, or subjective interests in the intervention; and (4) written informed consent. The exclusion criteria were as: (1) age<18years; (2) acute suicidality; (3) presence of a contraindication for IST, i.e., schizophrenic psychoses and dissociative disorders; (4) severe hearing impairment; (5) insufficient knowledge of German language; and (6) lack of capacity to consent.

All participants of the psycho-oncological intervention provided a written informed consent. The study complied with the Declaration of Helsinki and the Ethics Committee of the University Clinic Center Heidelberg approved the protocol (S-537/2017). We registered the study in the German Clinical Trials Registry (registration no. DRKS00013493).

### Intervention

Participants received one to four sessions of our supportive intervention using IST. The first session commenced before the mask was adjusted. The sessions were delivered by either a clinical psychologist or a physician (medical doctor during their residency training in internal medicine and psychosomatics) and lasted for about 50min. The therapists followed a manualized format for the sessions. The first session covered an anamnesis talk, short introduction to the rationale of imagination, guided imagination exercise [e.g., the inner safe place (Reddemann, 2005)], and debriefing. During the exercise “inner safe place,” patients are guided to seek out a safe place and explore it with all their senses. If necessary, we offered further sessions to intensify the guided imagination exercise. In these sessions, further imagination exercises were guided (e.g., the inner garden). Each participant received a CD or mp4 with imagination exercises.

### Measures

This trial aimed to test the feasibility of a screening and psycho-oncological intervention for patients undergoing radiotherapy with mask anxiety.

Feasibility and feasibility criteria of the screening were defined as:

- integration in clinical routine (feasibility of the screening as part of the registration),
- interdisciplinary workflow and cooperation (data transfer from the Department of Radiation Oncology to the psycho-oncological service),
- number of completed screenings,
- extent of psychological distress and demand for psycho-oncological support indicated by the screening (in the sense of a needs assessment), and
- reasons for non-participation in the intervention.

Feasibility and feasibility criteria of a psycho-oncological intervention using IST were defined as:

- utilization rate,
- evaluation of the participants (see evaluation form), and
- compliance to finish the radiotherapy.

Patients who participated in the intervention completed the following questionnaires after the first session of the intervention (t0) and after the last radiotherapy (t1):

We assessed patients’ distress on a 11-point numerical scale with endpoints of “no distress=1” or “extreme distress=11” using the NCCN Distress Thermometer (DT; Mehnert et al., 2006). It has been proven highly sensitive when evaluated against the established criteria. For its German version, a cut-off score of 5 has been recommended (Mehnert et al., 2006).

Depression symptoms were assessed using the Patient Health Questionnaire (depression module, PHQ-9; Lowe et al., 2004), a widely used screening tool in several clinical settings. This questionnaire evaluates the presence of nine symptoms of depressive episodes contained in the Diagnostic and Statistical Manual of Mental Disorders, fourth Revision. The PHQ-9 reveals good reliability, criterion, and construct validity and detects depressive symptoms and changes over time (Kroenke et al., 2001; Lowe et al., 2004; Martin et al., 2006). Higher values indicate more severe symptoms. A cut-off value between 8 and 11 screens for major depressive disorders (Manea et al., 2012). Cronbach’s *α* in our study was 0.79 (t0 and t1).

We assessed anxiety levels using the German GAD-7 (Spitzer et al., 2006), which is another reliable PHQ module to measure general anxiety symptoms, finding a good factorial and construct validity (Lowe et al., 2008). A cut-off value of ≥10 screens for anxiety disorders (Lowe et al., 2008). Cronbach’s *α* in our study was 0.90 (t0) and 0.88 (t1).

Health related quality of life (HRQOL) was assessed using the Short-Form Health Survey (SF-12), a generic questionnaire with good psychometric properties (Bullinger and Kirchberger, 1998) that allows multidimensional assessment of HRQOL in various disease groups (Ware et al., 1996). The SF-12 provides two subscales: mental component summary scores (MCS), which assess MQoL, and physical component summary scores (PCS), which assess PQoL. Scores range from 0 to 100 and higher scores indicate higher QoL. Cronbach’s *α* in our study was for PQoL 0.83 (t0) and 0.62 (t1), for MQoL 0.50 (t0) and 0.48 (t1).

For this study, a customized 5-point Likert evaluation scale (0=does not apply at all; 5=completely true) was used to investigate the subjective satisfaction and benefit of IST during radiotherapy (items are described in the results section). The evaluation scale was only assessed at t1.

### Statistics

Data were analyzed with IBM SPSS Statistics for Windows, version 24 (IBM Corporation, 2016). For investigation of the clinical (secondary) outcomes, we used Fishers Z and t-tests for independent samples to test the differences between the study participants and dropouts. We used paired t-tests to test the differences between t0 and t1 in the clinical measures. Effect sizes were calculated for repeated measures design, taking the correlation between t0 and t1 into account (Morris and DeShon, 2002), and interpreted by Cohen’s *d*; where *d*=0.2 is considered a small effect, *d*=0.5 is considered a medium effect, and *d*=0.8 is considered a large effect (Cohen, 1988). The reasons for non-participation were qualitatively analyzed by the CS.

## Results

### Screening

We screened a total of 1,020 patients. The mean distress by anxiety symptoms was 3.25 (median=2; standard deviations (*SD*)=2.62; range: 1–10). Two hundred and fifty-seven (25.2%) patients indicated a distress ≥5 and 141 (13.8%) patients reported panic attacks. Of the patients with heightened distress or panic attacks, 79 reported both a distress of ≥5 and panic attacks (7.9%). Five hundred and four (50%) patients were additionally asked for their subjective interest in the intervention. In this subpopulation, 25% reported a subjective interest, 65% reported no interest, and the remaining 10% was reported as missing value. In the group of interested patients, mean distress by anxiety was 3.69 (median=3; *SD*=2.74), 40 (31.7%) patients reported a distress ≥5, and 33 (26.2%) patients indicated panic attacks.

**Figure 1:**
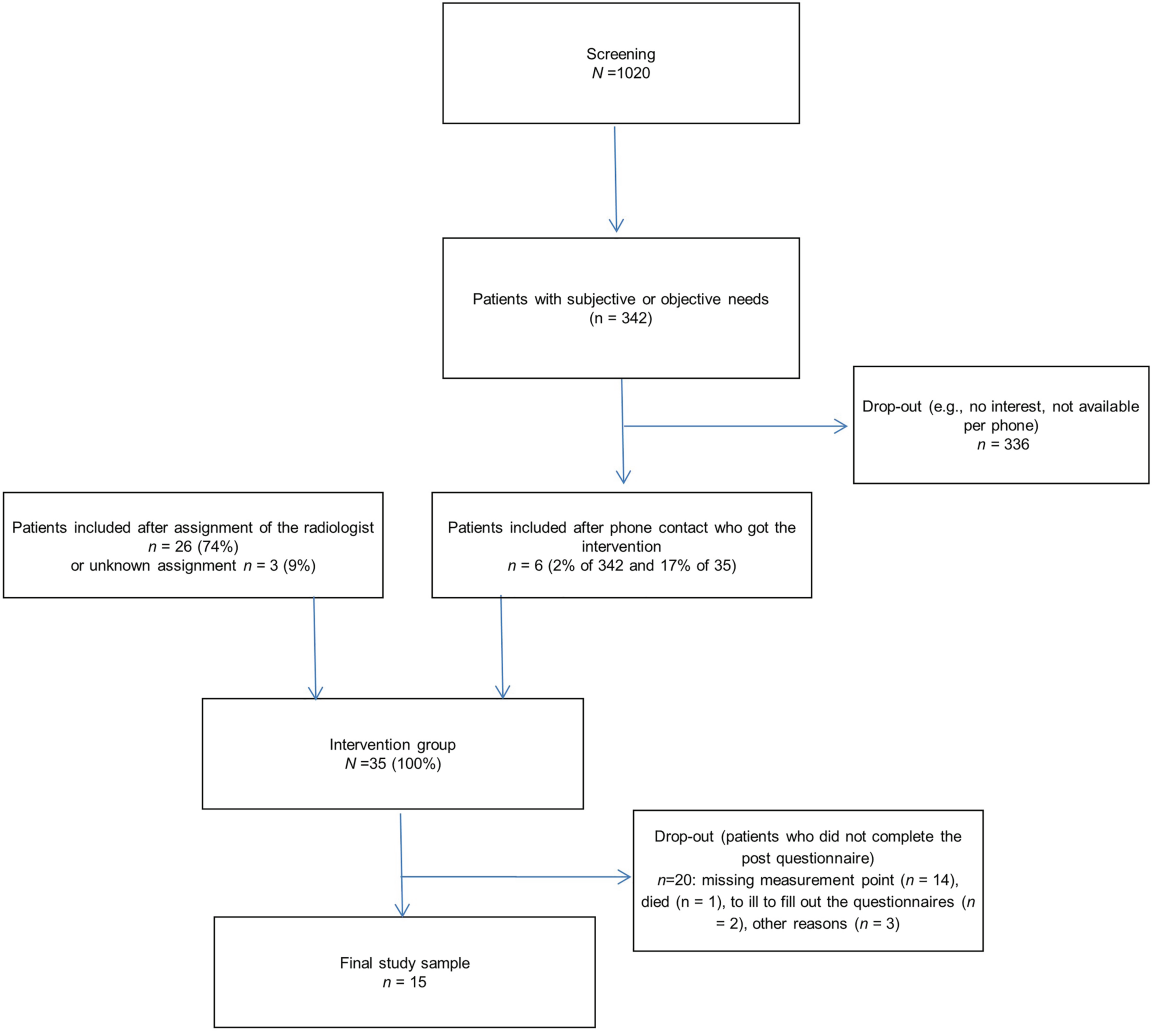
Study flow chart.

All patients with a distress score≥5 or subjective interest were contacted by a psycho-oncologist through phone and offered participation in the study (see Figure 1).

For *n*=77 patients with subjective interest (with or without objective needs) and *n*=163 patients with purely objective needs, we explored the reasons for non-participation. In patients with subjective interest, the reasons depict as: patient was not available per phone (*n*=16); no need/no interest (*n*=16); treatment not at the University Hospital Heidelberg (UKHD, *n*=10); patient gets already psychosocial support (*n*=4); interest in supportive counseling, but not in the intervention (*n*=4); no mask anxiety (*n*=4); no radiotherapy (n=3); physical problems (e.g., aphasia; *n*=3); and no specification or other reasons (*n*=17).

Among patients with purely objective needs, the main reason for non-participation was as: patient was not available per phone (*n*=38); no need/no interest (*n*=34); patient is not treated with radiotherapy (n=18); treatment not at the UKHD (*n*=12); radiotherapy already completed (*n*=9); cyberknife radiotherapy (*n*=9);^2^ no German language (*n*=8); patient gets already psychosocial support (*n*=6); and other reasons or no specification (*n*=29).

### Intervention

Thirty-five participants received the intervention with IST. 74% of the participants were assigned by a radiation oncologist. We collected data for the two measurement points from 15 participants (see Figure 1). Descriptive statistics of the study population are shown in Figure 1. We found no differences between participants who completed the questionnaire study (*n*=15) and participants who were excluded due to missing values or drop out (*n*=20) regarding patient and tumor-related factors (see Table 1). All but one participant (due to nausea) completed the radiotherapy.

**Table 1:** Descriptive statistics of the study population.

	study participants	other participants	Chi-Square or Fishers *Z*-test, *p*
	(*n* =15)*n* (%)	(*n* =20)*n* (%)	
sex			1.18, 0.321
female	7 (46.7)	13 (65.0)	
entity			
head and neck	5 (33.3)	9 (45.0)	4.20, 0.145
primary brain tumors	6 (40.0)	2 (10.0)	
brain metastases	4 (26.7)	9 (45.0)	
curative (yes)	10 (66.7)	8 (40.0)	2.44, 0.176
simultaneous chemotherapy	3	9	2.38, 0.163
	*M* (*SD*), (range)	*M* (*SD*), (range)	*t*-test, *p*
age	47.5 (13.8), (23, 71)	55.7 (12.9), (27, 72)	−1.80, 0.081
Karnofsky Performance Status	80 (9.2), (70, 100)^1^	83 (7.2), (70, 90)^2^	−0.86, 0.399
Radiotherapy Duration (in days)	35.5 (12.4), (1, 46)	30.9 (18.2), (1, 53)	0.89, 0.382
Number of Psychological Sessions	2.2 (0.9), (1, 4)	1.6 (0.8), (1, 3)^1^	0.87, 0.071

1 One missing value in this group;

2 Four missing values in this group.

Descriptive statistics and differences between patients who filled out the t0 and t1 questionnaire and patients with missing questionnaires or drop out (others). *M*=mean, *SD*=standard deviation.

Participants evaluated the intervention at t1 as positive (*M*=4.2, *SD*=0.78; range: 3–5) and helpful (*M*=3.73, *SD*=0.96; range: 2–5) and would recommend the intervention to others (*M*=4.3, *SD*=0.82; range: 2–5). They were able to familiarize with and apply the imagination exercises (*M*=3.73, *SD*=1.16, range: 1–5), could make use of IST during the radiotherapy treatment (*M*=3.53, *SD*=1.36, range: 1–5), and indicated a positive impact on regulating anxiety and worries during radiotherapy (*M*=3.53, *SD*=1.187, range: 2–5).

Results from the paired t-tests showed no significant pre-post differences in depression, anxiety, distress, MCS, and PCS, with small to medium effect sizes (see Table 2).

**Table 2:** Means (*M*) and standard deviations (*SD*) at T0 and T1, T- and p-scores, and effect sizes with 95% confidence interval.

	*n*	T0	T1	T/p-Wert	Cohen’s *d* (95% CI)
		*M* (*SD*)	*M* (*SD*)		
Depression (PHQ-9)	15	10.93 (4.85)	10.73 (4.80)	0.18/0.860	0.05 (−0.67, 0.76)
Anxiety (GAD-7)	15	9.47 (5.28)	8.53 (5.03)	0.71/0.492	0.18 (−0.54, 0.90)
Distress	13	7.92 (1.38)	7.00 (2.64)	1.45/0.172	0.67 (0.12, 1.46)
Psychological QoL (MCS; SF-12)	15	36.46 (7.55)	32.45 (6.40)	1.78/0.097	0.44 (0.29, 1.16)
Physical QoL (PCS; SF-12)	15	38.26 (10.41)	37.60 (8.50)	0.33/0.747	0.05 (−0.67, 0.77)

Note QoL=quality of life; GAD-7=anxiety scale of the PHQ-D; PHQ-9=depression scale of the PHQ-D; SF-12=short-form health survey; MCS=mental component score; PCS=physical component score; and CI=confidence interval.

## Discussion

To the best of our knowledge, this is the first study investigating a psycho-oncological care program for patients with HNC and brain malignancies before mask fixation and radiotherapy. Good feasibility was shown in (1) the high number of screened patients, thus demonstrating a good integration of the screening in the clinical routine as well as the willingness of the patients to complete the screening; (2) the good interdisciplinary workflow (implementation of the screening, data transfer from the department of radiotherapy to the psycho-oncologists); and (3) the assignment of the intervention from the radiologists in charge. All but one patient (due to nausea) completed the planned radiotherapy, which might indicate compliance-improvements for the radiotherapy due to the intervention. The low utilization rate among screened patients indicates barriers and implications for future psycho-oncological interventions for patients before radiotherapy with mask fixation.

### Screening

Results of our screening in patients with malignancies of the brain or head and neck before radiotherapy show that a quarter of patients suffer under distress due to anxiety and 14% suffer under panic attacks. This is in line with the data described in the literature (Pascoe et al., 2000; Semple et al., 2004; Ledeboer et al., 2005; Haman, 2008; Halkett et al., 2009; Semple et al., 2013) and underlines the importance of psycho-oncological support for these patients.

However, only 2% of patients with subjective interests or objective needs utilized the psycho-oncological support. Compared to studies with various cancer entities (Clover et al., 2015; Faller et al., 2017; Riedl et al., 2018), the utilization rate in our sample was rather low. Due to the high number of psychologically burdened patients in our sample, and the high distress elicited by the mask fixation, future studies are required to address the barriers of the utilization of psycho-oncological services in this patient group. Hence, the high number of patients with subjective interest in the intervention who eventually did not participate in the intervention, might indicate that only above-average motivated patients participated in our intervention. Future studies are needed to investigate potential selective effects.

The main barriers we could explore when contacted patients due to subjective or objective needs were no subjective need or no availability per phone. The second finding suggests a procedure where patients are directly addressed after they completed the screening.

The low number of participants (also among patients with subjective interest) might raise the question of the utility of the screening in this patient group. Distress screening is an economic way of detecting cancer patients with high distress. Therefore, we suggest a stronger involvement of the physicians in charge after patients have undergone the screening. The personal assignment by the radiation oncologist may increase the compliance and acceptance of psycho-oncological support and facilitates the accessibility to patients. For instance, in a multidisciplinary psychosocial stepped-care approach with various cancer entities, screening data were fed back to the clinicians in charge; the clinicians and patients discussed the data and decided if more psychosocial support was needed (Singer et al., 2019). Future studies should examine if this step (feedback to clinician and discussion) increases the utilization of psycho-oncological support in patients before mask fixation.

In this study, the high assignment rate by radiotherapy oncologists reveals their awareness of patients’ psychological distress and highlights the need and feasibility of psycho-oncological support. It underlines the importance of the sensibility of clinicians and staff to enquire about anxiety or psychological burden during consultations and initiate psychosocial support. Results from a needs assessment underline the wish of patients with head and neck tumors for more emotional support during and after the radiotherapy (Van Overveld et al., 2018). Therefore, integrated multidisciplinary care with a psychosocial screening as well as the involvement of psycho-oncologists is crucial to address the needs and burdens of patients (Van Overveld et al., 2017).

### Feasibility of IST Intervention

Participants experienced our intervention with IST, finding it helpful and effective. All measures remained at least statistically stable from pre- to post-treatment (radiotherapy). As other studies have shown an increase in psychological symptoms immediately at post-treatment (Stiegelis et al., 2004), these findings might indicate a positive effect of our intervention. Due to our small sample size and the small statistical power, our intervention could still be clinically relevant, although the results are not statistically significant. Effect sizes were the largest for distress reduction, which is in line with the brief intervention with IST that does not aim at a general improvement of psychopathology but a reduction of situational stress during radiotherapy treatment procedures. However, there was also considerable variation in individual effect sizes, which indicates that some patients profited more than others.

Two previous HNC case studies (Dabrowski and Grayer, 2018; Dinapoli et al., 2019) have shown that CBT and EMDR are promising approaches to reduce anxiety in patients with HNC, with possibly higher impact but higher effort and costs. Future studies should develop and examine economic and low-threshold psycho-oncological interventions for patients before mask fixation.

CBT approaches, mindfulness/relaxations techniques, and psycho-educational/skills interventions are recommended for patients with HNC (Williams, 2017). Blended CBT (in which face-to-face and online therapy are combined) is an (cost-)effective and promising approach for treating anxiety in cancer (Burm et al., 2019) and may increase the compliance and acceptance of support. As a result of this, we are planning an innovative randomized controlled trial to investigate a blended therapy program for patients before radiotherapy with mask fixation. In this approach, we plan to investigate the effects of supportive psycho-oncological sessions in combination with a cancer-specific app, which assesses patient-reported outcomes and provides cancer- and treatment-specific information, including psycho-education, relaxation, and mindfulness exercises.

Patients’ compliance to radiotherapy and psychological comfort during radiotherapy with mask fixation becomes more relevant, since stereotactic radiotherapies is used more frequently (Halasz et al., 2013; Alcorn et al., 2018; Barbour et al., 2020) and sessions can take 60min or longer. It is worthy to note that nearly all included patients completed the radiotherapy and that only one participant disrupted the therapy due to nausea. Owing to approximately 24% treatment disruption rates in other studies (Antonia et al., 2017), it might be indicative that our intervention has the positive effects of adherence of patients under radiotherapy. Repetitive session disruptions can lead to inefficient functioning of radiotherapy facilities, which is expensive and even more relevant, consume valuable treatment time in areas with limited access to medical resources. However, it might be that patients with high motivation to comply with the treatment were also about-average motivated to participate in our intervention. Further studies are needed to investigate potential selective effects. Another aspect that must be considered is that interventions require some preparation time for patients, resources, and trained staff. In emergencies or urgent situations, fast-acting medications, such as benzodiazepines, could be an adequate choice. However, in the study by Nixon et al. (2019), only 25% of the participants with mask anxiety found the medication helpful. Furthermore, if radiotherapy sessions are scheduled for over 7weeks, dependence potential of these substances should not be underestimated, thus instigating the need for adequate psycho-oncological interventions.

### Strengths and Limitations

The strengths of this study include the large, screened cohort with patients at different stages in the course of the disease, the standardized psychological assessment, and the innovative psycho-oncological care approach. Even though the exact number of patients who receive radiotherapy with mask fixation is not recorded as standard and is not available, it can be assumed that the vast majority of patients with mask fixation was screened (over 5000 patients were treated with radiotherapy in this period and approximately 20% of patients received radiotherapy with mask fixation). The inclusion of all patients facing mask fixation depicts another strength, since it has mainly been investigated in patients with HNC till date. The investigation of barriers is helpful for future studies in this patient group. However, the lack of a control group makes it difficult to interpret our results over and above the positive findings concerning feasibility. The deployed questionnaires were rather broad and less focused on the experience of the radiotherapy intervention situation itself. Many patients were not available per phone, so that their needs could not be clarified. Future studies are required, in addition to randomized controlled trials and specific outcomes measures, to investigate the effectiveness of psycho-oncological care approach in patients with HNC or BC before mask fixation.

## Conclusion

The present study indicates a good feasibility and need for a psycho-oncological care program for patients with head and neck/brain malignancies before radiotherapy with mask-fixations. The screening results demonstrated the demand of psycho-oncological support for patients with head and neck/brain malignancies before radiotherapy with mask-fixations. However, since the utilization of our intervention was low, future studies are required to reduce the barriers and improve compliance to psycho-oncological services in this patient group. The involvement of the radiotherapy oncologist in charge might be promising to increase the accessibility of patients, and the compliance and acceptance of psycho-oncological interventions. We experienced a good feasibility of a psycho-oncological care program for patients facing mask fixation and show the possible implications for psycho-oncological interventions and future approaches.

## Data Availability Statement

The raw data supporting the conclusions of this article will be made available by the authors, without undue reservation.

## Ethics Statement

The studies involving human participants were reviewed and approved by ethics committee University Heidelberg (S-537/2017). The patients/participants provided their written informed consent to participate in this study.

## Author Contributions

SA, CS, LL, JE, JD, and IM: conceptualization. SA and CS: data curation. CS: formal analysis. SA and IM: funding acquisition. CS, LL, SR, PW, KZ, CF, JM, JK, RS, JH-R, LK, SA, KL, TH, and SR: investigation. SA, CS, CN, and IM: methodology. SA and CS: project administration. JD and H-CF: resources. SA, CS, and IM: supervision. SA, CS, and IM: writing – original draft. SA, CS, BF, and IM: writing – review & editing. All authors have read and agreed to the published version of the manuscript.

## Funding

The study was financially supported by the NCT Heidelberg.

## Conflict of Interest

SA and JD received grants from the Accuray International Sàrl and the Merck Serono GmbH outside the submitted work. SA received grants from the Novocure GmbH, MSD and Astra Zeneca outside the submitted work, the Novocure GmbH, Actinium Pharmaceuticals, Telix Pharmaceuticals shareholding, and Advisory Board Sanofi Genzyme and Accuray Incorporated. JD received grants from the Clinical Research Institute GmbH (CRI), View Ray Inc., Accuray Incorporated, RaySearch Laboratories AB, Vision RT limited, Astellas Pharma GmbH, Astra Zeneca GmbH, Solution Akademie GmbH, Ergomed PLC Surrey Research Park, Siemens Healthcare GmbH, Quintiles GmbH, Pharmaceutical Research Associates GmbH, Boehringer Ingelheim Pharma GmbH Co, PTW-Freiburg Dr. Pychlau GmbH, and Nanobiotix AA outside the submitted work.

The remaining authors declare that the research was conducted in the absence of any commercial or financial relationships that could be construed as a potential conflict of interest.

## Publisher’s Note

All claims expressed in this article are solely those of the authors and do not necessarily represent those of their affiliated organizations, or those of the publisher, the editors and the reviewers. Any product that may be evaluated in this article, or claim that may be made by its manufacturer, is not guaranteed or endorsed by the publisher.

## Footnotes

^1^In line with the cut-off of the NCCN Distress Thermometer, we set the cut-off at â‰¥5.

^2^Originally, cyberknife radiotherapy was defined as an exclusion criterion. Due to the high number of patients with cyberknife therapy, we changed the study protocol during the trial.
